# A Comparison of Contractile Properties and Acute Muscle Fatigue Response in Adult Females with Non-Specific Chronic Low Back Pain

**DOI:** 10.3390/bioengineering11121202

**Published:** 2024-11-28

**Authors:** Hyungwoo Lee, Seungwon Lee, Chanki Kim, Kyoungkyu Jeon

**Affiliations:** 1 Division of Sport Science, Incheon National University, 119 Academy-ro, Yeonsu-gu, Incheon 22012, Republic of Korea; hyungwoolee@inu.ac.kr (H.L.); kimchangi960430@inu.ac.kr (C.K.); 2 Department of Human Movement Science, Incheon National University, 119 Academy-ro, Yeonsu-gu, Incheon 22012, Republic of Korea; 3 Functional Rehabilitation Biomechanics Laboratory, Incheon National University, 119 Academy-ro, Yeonsu-gu, Incheon 22012, Republic of Korea; monkeylee97@inu.ac.kr; 4 Health Promotion Center, Incheon National University, 119 Academy-ro, Yeonsu-gu, Incheon 22012, Republic of Korea; 5 Sport Science Institute, Incheon National University, 119 Academy-ro, Yeonsu-gu, Incheon 22012, Republic of Korea

**Keywords:** acute muscle fatigue response, erector spinae, muscle contractile properties, non-specific low back pain, tensiomyography, trunk strength

## Abstract

This study examined the erector spinae contractile properties, trunk isokinetic strength, and differences in acute muscle fatigue response after exercise in young females with and without non-specific chronic low back pain (NSCLBP). This study evaluated participants using tensiomyography and isokinetic trunk strength tests. An independent *t*-test compared the control group and the NSCLBP group, while a two-way mixed ANOVA analyzed differences in the erector spinae’s acute muscle fatigue response before and after exercise within and between groups. The results of the tensiomyography indicated that the NSCLBP group exhibited significantly lower Dm and Vc (*p* < 0.05) compared to the control group, while Tc showed no significant difference between groups. Significant differences in all variables were observed between the groups in the isokinetic trunk strength test (*p* < 0.05). Furthermore, the two-way mixed ANOVA revealed significant group main effects in Dm and Vc of the erector spinae (*p* < 0.05). This study found that non-specific chronic low back pain is linked to a decrease in Dm, Vc, and trunk isokinetic strength in both extensor and flexor muscles. It suggests that future research should further investigate the acute muscle fatigue response in individuals with and without NSCLBP.

## 1. Introduction

Low back pain (LBP) is one of the leading causes of musculoskeletal disorders in the general population [1]. Recurrence of LBP is very common, with over 60% of LBP patients developing chronic low back pain (CLBP) [2,3,4]. Approximately 85% of all CLBP cases are classified as non-specific chronic low back pain (NSCLBP), where a specific pathological cause is challenging to identify [5,6,7,8,9,10]. Despite numerous studies aimed at understanding the fundamental mechanism of NSCLBP, appropriate treatment and management are not achieved due to the lack of a clear pathological cause and diagnosis [11,12]. Additionally, because various factors cause NSCLBP, limited treatment and intervention methods can lead to negative outcomes such as disability, limited mobility, poor health, decreased quality of life, and depression [13,14].

NSCLBP is characterized by tightness and stiffness in the muscles surrounding the lumbar spine, which are essential for maintaining spinal stability [15]. In individuals with NSCLBP, reciprocal atrophy of the lumbar muscles results in poor muscular endurance [16]. Furthermore, individuals with NSCLBP exhibit lower levels of muscle activation and movement variability compared to healthy individuals during acute muscle fatigue induced by repetitive movements. They also demonstrate lower coping abilities, such as endurance and resistance, when muscle fatigue is induced [17]. Increased muscle fatigue can reduce muscle contraction efficiency, leading to changes in peripheral nerves or dysfunction in the muscles, which do not properly recruit motor neurons in the central nervous system. Consequently, this delay in muscle response time increases stress on the spine [18,19]. Dysfunction in the neuromuscular system has been associated with increased spinal instability, tissue tightness, and stiffness, all of which contribute to the persistence of pain in NSCLBP [16,20].

Hence, it is imperative to the contractile properties of erector spinae and the acute muscle fatigue response to develop suitable intervention strategies for individuals with NSCLBP. Nevertheless, the majority of the previous studies have utilized electromyography as the primary equipment, which is dynamic and assesses electrical activity during voluntary muscle contractions, making it challenging to pinpoint the physiological mechanisms underlying responses to fatigue [21]. According to previous studies, static functional tests of the erector spinae in LBP patients are recommended to address the limitations of dynamic functional tests for LBP patients. For this purpose, tensiomyography (TMG) is reported to be used for non-invasively measuring acute muscle fatigue following acute exercise, with studies indicating that the superficial layers of skin, subcutaneous fat, and muscle fascia have no significant effect on TMG measurements [22,23,24].

This study aims to compare and analyze the erector spinae contraction properties and trunk isokinetic strength in young women in their 20s with and without NSCLBP. Additionally, this study aimed to examine the differences in the acute muscle fatigue response of the erector spinae following acute exercise.

## 2. Materials and Methods

### 2.1. Participants

For this research, a control group (n = 21) comprising individuals without back pain symptoms in the preceding 3 months and a group with NSCLBP (n = 21) were selected. The NSCLBP group included participants who could not determine the specific pathological cause of the musculoskeletal and neurological disease with a Visual Analogue Scale (VAS) score of 5 or higher, indicating moderate disability due to LBP [25]. Exclusion criteria involved individuals with musculoskeletal or nervous system disorders and those diagnosed with specific chronic low back pain with specific pathological causes such as neuromyopathy and malignancies. This study received approval from the Institutional Review Board of Incheon National University (INUIRB No. 7007971-202108-005) and was carried out after providing a comprehensive explanation of the study’s content and procedures to the participants, who then consented to take part. Participant’s back pain levels were assessed using a VAS, and the level of functional disability due to LBP was evaluated using the Korean Oswestry Disability Index (KODI) [25,26]. The physical characteristics of the participants in this study are presented in Table 1.

### 2.2. Tensiomyography

**Figure 1:**
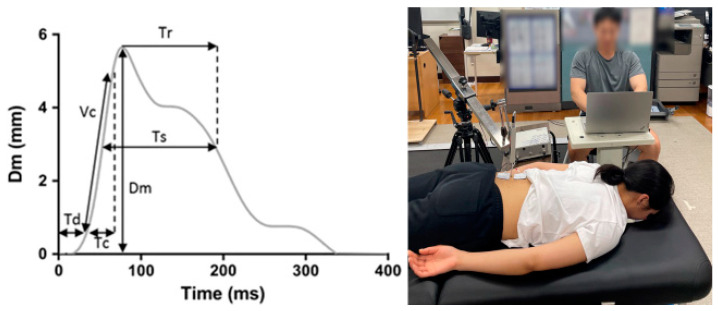
Measurement of tensiomyography.

In this study, the TMG-100 System (Electrostimulator, Ljubljana, Slovenia) was used to analyze the contractile properties of the erector spinae (Figure 1) [27]. TMG uses single electrical stimulation ranging from 0 to 100 mA to induce involuntary muscle contractions; this method allowed for a non-invasive evaluation of neuromuscular characteristics such as single muscle stiffness, contraction velocity, muscle fiber type, and muscle fatigue [28,29,30]. TMG is recognized as a reliable and highly sensitive technique for assessing LBP, overall muscle function, and peripheral fatigue [23,31,32]. Furthermore, TMG has been suggested as a monitoring tool to quantify and identify the effects of peripheral fatigue, including muscle fatigue and damage, as well as changes in mechanical capacity and performance following various types of exercise [33,34].

In this research, participants were instructed to refrain from consuming caffeine, engaging in physical exercise, and utilizing myofascial release techniques in the 24 to 48 h leading up to the TMG measurements, as these factors have the potential to influence the outcome variables [35,36]. The TMG assessments were conducted following a 5 min rest period to ensure relaxation of the erector spinae. A cushion was positioned under the ankle in a prone position to maintain a 5° flexion of the knee joint, with an additional wedge cushion anteriorly to stabilize the lumbar lordosis, positioned at the anterior superior iliac spine. The anatomical location of the erector spinae was determined using the anatomical guide method described by Perotto et al. (2011) [37]. Subsequently, a digital displacement TMG sensor (GK 40, Panoptik d.o.o., Ljubliana, Slovenia) was vertically placed at the designated measurement site, considering a maximum radial displacement (Dm) value of 15 mm. Electrode pads consisting of two electrodes (50 × 50, T.Y. Sherry International Co., Ltd., Taiwan, China) were attached [38]. The electrical stimulation intensity commenced at 20 mA, following the protocol of previous studies, and was incrementally increased by 20 mA until the maximum displacement value was achieved. A 15 s rest period was implemented between measurements to mitigate muscle fatigue and post-activation potentiation effects [36,39]. All TMG assessments were performed by a single evaluator with over 2 years of experience.

### 2.3. Isokinetic Trunk Strength Test and Acute Muscle Fatigue-Induced Intervention

**Figure 2:**
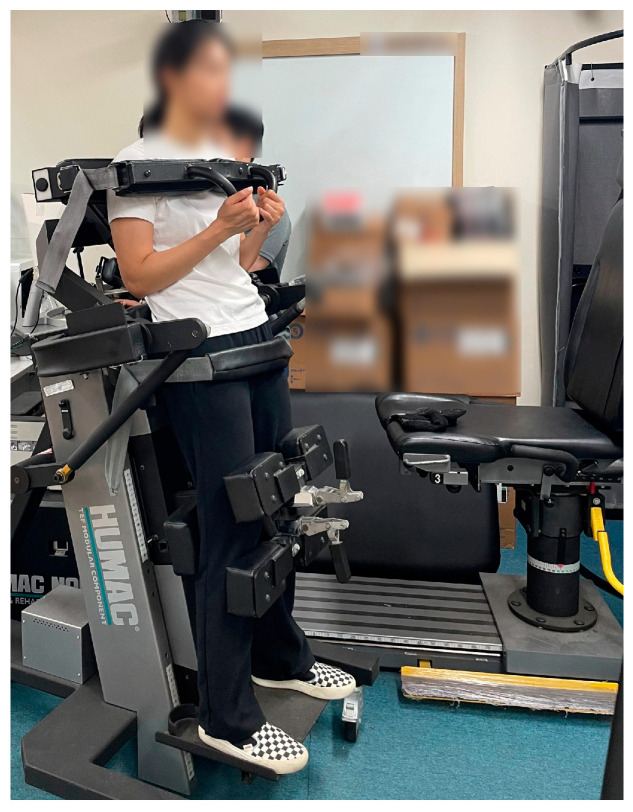
Measurement of isokinetic trunk strength.

In this study, Humac Norm Testing and Rehabilitation (CSMi Medical & Solution, Stoughton, MA, USA) was used to measure the isokinetic strength of the trunk and to induce an acute fatigue response of the erector spinae (Figure 2). This equipment was chosen for its ability to avoid inducing exacerbating symptoms of LBP post-measurement, as well as for its consistency and excellent reliability (Intraclass Correlation Coefficient; ICC = 0.85–0.98) in assessing trunk muscle function in patients with back pain [40,41].

**Figure 3:**
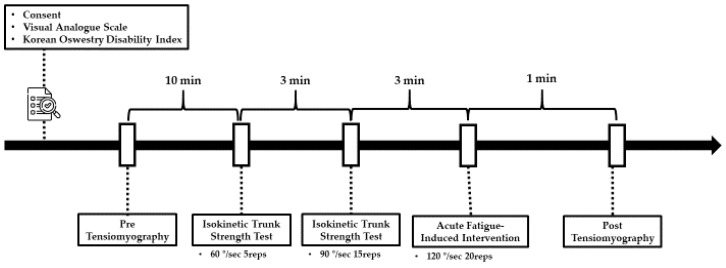
Outline of experimental protocol.

Prior to the measurements, all participants engaged in dynamic stretching exercises, lasting 10 min, to reduce the risk of injuries. The range of motion for maximum trunk flexion and extension was set within a pain-free range for all subjects to prevent adverse effects and exacerbation of symptoms due to excessive movements. Measurements were performed 5 times at an angular velocity of 60°/s and 15 times at an angular velocity of 90°/s, with a 3 min rest interval between measurements to minimize muscle fatigue. Following the isokinetic trunk strength measurements at 60°/s and 90°/s, participants underwent 20 repetitions at 120°/s to induce acute fatigue in the erector spinae. All measurements were carried out by a single evaluator to ensure consistency and psychological factors such as motivation were standardized across all participants. All measurements were conducted as shown in Figure 3.

### 2.4. Data Processing

#### 2.4.1. Contractile Properties of the Erector Spinae and Response Changes in Acute Muscle Fatigued

Reliable and clinically relevant variables obtained from the TMG measurements were utilized to compare and analyze the static contractile properties of the erector spinae among different groups and to assess the acute fatigue response of the erector spinae within and between groups following acute exercise. These variables included Tc (ICC = 0.70–0.98), which signifies the duration for the muscle to contract from 10% to 90% of its maximum contraction displacement, and Dm (ICC = 0.91–0.99), which measures the maximum contraction displacement of the muscle [31,42,43]. Furthermore, the velocity of contraction (Vc), a practical and highly reliable variable (ICC > 0.95), was calculated to evaluate the mechanical function of muscles using Formula (1) below [31,44]. To conduct a comparative analysis of measurement variables within and between groups, the average values of the right and left sides of the erector spinae were compared and analyzed.

(1) $$Vc\left(mm/ms\right)=Dm/(Tc+Td)$$

#### 2.4.2. Isokinetic Strength of Trunk

To ensure a precise comparison and analysis of isokinetic strength characteristics, the absolute maximum torque value (peak torque, Nm) of the trunk flexors and extensors was determined. Subsequently, the relative maximum torque value (BW, %) was calculated by incorporating the subject’s weight into the calculation, as outlined in Formula (2), for further analysis [45]. Furthermore, the strength balance ratio between the trunk flexors and extensors was assessed by calculating the strength ratio (F/E ratio) of the flexors and extensors using Formula (3) below.

(2) $$BW\left(\%\right)=Peak\;Torque/Body\;Weight$$

(3) $$Ratio\left(\%\right)=(Flexor\;Value/Extensor\;Value)\times 100$$

### 2.5. Statistical Analysis

All data in this study were presented as mean and standard deviation (Mean ± SD) and analyzed using SPSS 28.0 (IBM, Chicago, IL, USA), a statistical software for Windows. The normality of the data for all measured variables was assessed using the Shapiro–Wilk test, confirming normal distribution. Independent *t*-tests were then conducted to compare and analyze participant characteristics, static contractile properties of the erector spinae, and isokinetic strength characteristics of trunk flexors and extensors between groups. To examine the differences in the acute fatigue response of the erector spinae within and between groups before and after acute exercise, a two-way mixed ANOVA (2 × 2: Group × Time) was performed. In cases where an interaction effect was observed, post hoc analysis was conducted using Bonferroni’s multiple-comparison test. The significance level for all statistical analyses was set at *p* < 0.05.

## 3. Results

### 3.1. Static Contractile Properties of Erector Spinae

Upon examination of the static contractile properties of the erector spinae, no significant difference in Tc was observed between groups (t_40_ = −0.731; *p* = 0.471). However, significant variations between groups were identified in Dm (t_40_ = 2.709; *p* = 0.010) and Vc (t_40_ = 2.742; *p* = 0.010) (Table 2).

### 3.2. Isokinetic Trunk Strength Test

As a consequence of the isokinetic trunk strength test (60°/s) analysis, significant variances were observed between groups in the relative maximum torque values (%) of the flexor and extensor (t_40_ = 2.446; *p* = 0.020, t_40_ = 4.684; *p* < 0.001) (Table 3).

The isokinetic trunk strength test (90°/s) revealed significant variations between groups in the relative maximum torque values (%) of the flexor and extensor (t_40_ = 2.567; *p* = 0.015, t_40_ = 5.697; *p* < 0.001), as presented in Table 3.

Upon examination of the strength ratio (F/E ratio) of the flexor and extensor, a notable variance was observed between the groups during the isokinetic trunk strength test at 60°/s and 90°/s (t_40_ = −3.751; *p* < 0.001, t_40_ = −4.496; *p* < 0.001) (Table 3).

### 3.3. Response Changes in Acute Fatigued Muscles

The analysis conducted using a two-way mixed ANOVA on the acute muscle fatigue response based on the TMG variable revealed a significant group main effect for the Dm variable [F_(1,40)_ = 7.939; *p* = 0.007; np^2^ = 0.166]. Additionally, a significant group main effect was observed for the Vc variable [F_(1,40)_ = 6.733; *p* = 0.013; np^2^ = 0.144] (Table 4).

## 4. Discussion

This study aimed to compare and analyze the static contractile properties of the erector spinae and the isokinetic strength of the trunk flexor and extensor muscles in young adult women in their 20s with and without NSCLBP. Additionally, this research sought to examine the differences in acute muscle fatigue response of the erector spinae following acute exercise within and between the groups.

In the results of static contractile properties of the erector spinae, we found no significant difference in Tc between the two groups, but Dm and Vc were significantly lower in the NSCLBP group compared to the control group. The decrease in lumbar muscle function is linked to the development and persistence of NSCLBP, leading to reductions in anatomical muscle cross-sectional area, increased intramuscular fat content, muscle atrophy, and a shift towards Type I muscle fibers [46,47]. While Tc, which is highly correlated with Type I muscle fibers, did not differ significantly between the groups in this research, it was observed that the NSCLBP group tended to have higher values than the control group [22]. These findings align with investigations on muscle fiber types in NSCLBP using biopsy samples, indicating an increase in oxidative muscle fibers (Type I) and a decrease in glycolytic muscle fibers (Type II) [48]. Further research is necessary for the prevention and management of NSCLBP, as inconsistencies in previous studies have made it challenging to determine the presence of muscle fiber changes in NSCLBP [49,50].

According to a previous study, both Dm and Vc were found to be significantly lower in the NSCLBP group compared to the control group, which aligns with the results of the present study [51]. Dm typically shows lower values due to increased tightness and stiffness of the muscle [27]. It has been reported that significant differences in Dm can indicate variations in the muscle’s recovery capacity and the extent of damage [22]. Furthermore, a decrease in Vc has been linked to muscle overuse and fatigue [52]. Previous research has indicated a 20% increase in the stiffness of the erector spinae in NSCLBP patients, suggesting that LBP contributes to alterations in superficial muscle structures like the erector spinae, potentially leading to chronic pain [53,54]. It has also been reported that individuals with LBP exhibit higher erector spinae activity compared to healthy individuals, and prolonged excessive activity of the erector spinae may result in lumbar dysfunction [55]. Additionally, deteriorating muscle condition, a contributing factor to NSCLBP, imposes continuous strain on the lumbar muscles, leading to increased muscle fatigue and NSCLBP [56]. Consequently, based on these findings, it can be inferred that the NSCLBP group would demonstrate lower Dm and Vc values than the control group.

As a result of the analysis of trunk isokinetic strength measurements, the NSCLBP group exhibited significantly lower relative isokinetic strength of trunk flexors and extensors compared to the control group at angular velocities of 60°/s and 90°/s. Moreover, the NSCLBP group demonstrated a significantly higher flexor and extensor strength ratio (F/E ratio) than the control group. Chronic pain has been found to impact muscle structure, including muscle atrophy, changes in muscle fibers, and intramuscular fat infiltration, as well as muscle function, such as strength and muscular endurance [57]. Reduced strength and imbalance of trunk flexors and extensors have been associated with the occurrence and severity of NSCLBP symptoms and contribute to its progression [41,57,58]. The relationship between pain, muscle structure, and muscle function is complex and bidirectional, influencing the persistence and recurrence of NSCLBP [57,59,60]. This study also measured the erector spinae using TMG and found that the NSCLBP group had significantly higher stiffness and slower muscle contraction velocity due to muscle fatigue compared to the control group. This aligns with findings on the biomechanical characteristics of the lumbar extensor fascia, where individuals with chronic low back pain exhibit higher tightness and stiffness and lower elasticity than healthy individuals due to fibrosis and decreased tissue flexibility [15,61]. These results suggest dysfunction in fascial mechanics, leading to inadequate fluid exchange, altered blood circulation, ischemia, muscle fiber deterioration, increased collagen production, and fascial fibrosis [62]. The elasticity of fascia influences the amount of energy it can store, impacting muscle strength [63]. Therefore, increased tightness and stiffness and decreased elasticity of the lumbar fascia restrict energy storage, resulting in muscle weakness, reduced muscle strength, endurance, and range of motion related to lumbar movements [64,65]. Overall, the NSCLBP group exhibited a lower relative isokinetic strength of trunk flexors and extensors and a higher F/E ratio compared to the control group, based on these findings.

The outcomes of the two-way mixed-design analysis of variance conducted on the acute muscle fatigue response of the erector spinae induced by acute exercise, categorized by the presence or absence of NSCLBP, indicated that the NSCLBP group exhibited significantly lower outcomes in Dm and Vc compared to the control group. Nevertheless, there were no significant main effects for time or group × time interaction effects, except for Dm and Vc. One of the primary attributes of NSCLBP is elevated muscle tightness and stiffness, alongside delayed muscle activity [66,67]. Consequently, it is posited that the main effect for the group manifested in Dm and Vc among the TMG variables. However, there were no significant main effects for time or group × time interaction effects. As per prior research exploring muscle fatigue during repetitive trunk flexion–extension, the neuromuscular system demonstrates compensatory adjustments such as modified muscle activation and movement coordination patterns to maintain optimal exercise performance [68]. Furthermore, one of the compensatory responses to prolonged muscle fatigue involves the lumbar spine directly bearing a load instead of the surrounding lumbar muscles, which may result in decreased muscle activation [69]. Based on these findings, it is considered that the isokinetic muscle testing protocol for trunk flexor and extensor muscles used in this study to induce acute fatigue may not have elicited an acute muscle fatigue response in the erector spinae due to compensatory responses. Additionally, it is judged that the isokinetic muscle testing protocol used in this study may have difficulty in inducing localized muscle fatigue responses, as it can involve multiple synergistic muscles, not only the erector spinae. Consequently, future investigations will necessitate further analysis to scrutinize the acute fatigue response based on the presence or absence of NSCLBP by implementing a protocol capable of readily inducing a localized muscle fatigue response.

## 5. Conclusions

The findings of this research suggest that the NSCLBP group displayed lower Dm and Vc, as well as reduced trunk isokinetic strength in the extensor and flexor muscles, compared to the control group. The results also indicate a potential imbalance between the flexor and extensor muscles. Future research may benefit from additional studies utilizing protocols that can effectively induce acute muscle fatigue to further investigate acute muscle fatigue responses to the presence or absence of NSCLBP. Additionally, based on the results of this study, if used as foundational data for developing exercise interventions for NSCLBP patients, it could have a positive impact on potential treatment methods for NSCLBP.

## Figures and Tables

**Table 1:** Characteristics of participants.

Variables	Control (n = 21)	NSCLBP ^a^ (n = 21)	*t*	*p*
Age (years)	23.05 ± 2.75	22.10 ± 1.51	1.391	0.172
Height (cm)	161.52 ± 5.15	163.07 ± 4.14	−1.069	0.291
Weight (kg)	53.76 ± 7.07	54.75 ± 7.62	−0.435	0.666
VAS ^b^ (cm)	0.71 ± 1.15	6.86 ± 0.85	−19.695	≤0.001 ***
KODI ^c^ (%)	3.39 ± 3.42	34.92 ± 7.92	−16.748	≤0.001 ***
Pain period (Month)	0.00 ± 0.00	49.24 ± 38.67	−5.836	≤0.001 ***

Data are Means ± Standard Deviation, *** *p* < 0.001. Abbreviations. ^a^: Non-specific chronic low back pain; ^b^: Visual Analogue Scale; ^c^: Korean Oswestry Disability Index.

**Table 2:** Results of tensiomyography of muscles of the participants.

Muscle	Variables	Control	NSCLBP ^a^	*t*	*p*
ES ^b^	Tc ^c^	15.43 ± 1.40	15.98 ± 3.09	−0.731	0.471
	Dm ^d^	3.82 ± 1.03	2.64 ± 1.73	2.709	0.010 *
	Vc ^e^	0.10 ± 0.02	0.07 ± 0.05	2.742	0.010 *

Note. Means ± Standard Deviation, * *p* < 0.05, Abbreviations. ^a^: Non-specific chronic low back pain; ^b^: erector spinae; ^c^: contraction time; ^d^: maximum radial displacement; ^e^: velocity of contraction.

**Table 3:** Results of isokinetic trunk strength of the participants (60°/s, 90°/s).

Variables			Control	NSCLBP ^a^	*t*	*p*
60°/s	Flexor	BW ^b^ (%)	232.05 ± 48.06	181.05 ± 82.60	2.446	0.020 *
	Extensor	BW ^b^ (%)	193.19 ± 52.96	108.90 ± 63.21	4.684	<0.001 ***
90°/s	Flexor	BW ^b^ (%)	234.38 ± 45.57	186.19 ± 72.96	2.567	0.015 *
	Extensor	BW ^b^ (%)	174.05 ± 49.53	89.62 ± 46.46	5.697	<0.001 ***
Ratio ^c^	60°/s		123.86 ± 24.43	186.95 ± 73.11	−3.751	0.001 **
	90°/s		140.62 ± 31.06	234.38 ± 90.39	−4.496	<0.001 ***

Note. Means ± Standard Deviation, * *p* < 0.05, ** *p* < 0.01, *** *p* < 0.001. Abbreviations. ^a^: Non-specific chronic low back pain; ^b^: relative maximum torque value; ^c^: flexor/extensor ratio.

**Table 4:** Results of two-way mixed ANOVA for the TMG variables.

	Group	Pre	Post	Group			Time			Group × Time		
				*F*	*p*	*n_p_* ^2^	*F*	*p*	*n_p_* ^2^	*F*	*p*	*n_p_* ^2^
Tc ^b^	Control	15.43 ± 1.40	15.86 ± 2.02	0.362	0.551	0.009	0.426	0.518	0.011	0.118	0.733	0.003
	NSCLBP ^a^	15.98 ± 3.09	16.11 ± 3.20									
Dm ^c^	Control	3.82 ± 1.03	3.63 ± 1.17	7.939	0.007 **	0.166	0.926	0.342	0.023	0.290	0.593	0.007
	NSCLBP ^a^	2.64 ± 1.73	2.58 ± 1.38									
Vc ^d^	Control	0.11 ± 0.03	0.10 ± 0.03	6.733	0.013 **	0.144	1.106	0.299	0.027	2.092	0.156	0.050
	NSCLBP ^a^	0.08 ± 0.05	0.08 ± 0.04									

Note. Means ± Standard Deviation, ** *p* < 0.01. Abbreviations. ^a^: Non-specific chronic low back pain; ES; erector spinae; ^b^: contraction time; ^c^: maximum radial displacement; ^d^: velocity of contraction.

## Data Availability

Data are not publicly available due to privacy.
